# A Molecular Analysis of Cytokine Content across Extracellular Vesicles, Secretions, and Intracellular Space from Different Site-Specific Adipose-Derived Stem Cells

**DOI:** 10.3390/ijms23010397

**Published:** 2021-12-30

**Authors:** Jerran Santos, Penelope V. Dalla, Bruce K. Milthorpe

**Affiliations:** Advanced Tissue Engineering and Stem Cell Biology Group, School of Life Sciences, University of Technology Sydney, Broadway, P.O. Box 123, Sydney 2007, Australia; Penelope.Dalla@health.nsw.gov.au (P.V.D.); Bruce.Milthorpe@uts.edu.au (B.K.M.)

**Keywords:** adipose-derived stem cells, extracellular vesicles, secretions, cytokines, multiplex assay, interaction networks

## Abstract

Cytokines are multifunctional small proteins that have a vital influence on inflammatory states of tissues and play a role in signalling and cellular control mechanisms. Cytokine expression has primarily been viewed as a form of direct secretion of molecules through an active transportation; however, other forms of active transport such as extracellular vesicles are at play. This is particularly important in stem cells where signalling molecules are key to communication managing the levels of proliferation, migration, and differentiation into mature cells. This study investigated cytokines from intracellular content, direct cellular secretions, and extracellular vesicles from adult adipose-derived stem cells isolated from three distinct anatomical locations: abdomen, thigh, and chin. The cells were cultured investigated using live cell microscopy, cytokine assays, and bioinformatics analysis. The cytokines quantified and examined from each sample type showed a distinct difference between niche areas and sample types. The varying levels of TNF-alpha, IL-6 and IL-8 cytokines were shown to play a crucial role in signalling pathways such as MAPK, ERK1/2 and JAK-STAT in cells. On the other hand, the chemotactic cytokines IL-1rn, Eotaxin, IP-10 and MCP-1 showed the most prominent changes across extracellular vesicles with roles in noncanonical signalling. By examining the local and tangential roles of cytokines in stem cells, their roles in signalling and in regenerative mechanisms may be further understood.

## 1. Introduction

Cytokines are a sizeable group of pleotropic small proteins that are produced by a range of cells [1]. They are generally well known for their role in the inflammatory response and control in the immune system [2]. They are also now recognised to be responsible for coordinating signalling and molecular processes across varied tissues and cell types of which the finer mechanisms are still being unravelled [3]. The secretion of cytokines from nonimmune cells has displayed functional roles in a variety of development stages, acting as trophic factors initiating the repair and regeneration of cells [4]. This is particularly important in stem-cell research as their expression and secretion have been explored in various capacities in mesenchymal stem or stromal cells [5,6]. Their role in adipose-derived stem cells (ADSCs) is slowly being elucidated and has received increased attention in recent research to understand their role in basal expression and during the differentiation process, as well as their possible therapeutic potential [6,7]. ADSCs have the inherent ability to spontaneously produce cytokines either to modulate their surrounding tissue or in response to stimuli [5]. Significant expression changes are often attributed to inflammation or disease states where secretions of IL-1β, IL-6, and TNF-α change dramatically in proinflammatory states [2]. The counter effect in anti-inflammatory states is seen by the increased expression of IL-4 and IL-10 [2]. Subtle variations in concentrations and combination changes can have profound effects in cells response to a stimulus, ranging from restoring the local site to a state of equilibrium or driving changes across entire populations of cells by guiding signalling and differentiation [8].

During stem-cell differentiation states, cytokines display functional roles in a variety of development stages, such as acting as trophic factors initiating the repair and regeneration of cells [9]. Furthermore, certain groups of cytokines, such as chemokines, regulate the directed growth and communication between migrating cells, which instigate direct cell-to-cell communication and contact signalling [3].

Cytokines cannot cross the cell’s lipid bilayer to enter the cytoplasm; their main mode of action is enacted through receptors at the cell surface and nucleus. Alternate routes and modes of cytokine delivery and translocation have been investigated including active transport. There is mounting evidence that various molecules, including arrays of proteins, peptides, and cytokines, are selectively packaged into extracellular vesicles (EVs) [10,11,12]. The EV cytokine cargo can be delivered to the intracellular space by membrane and EV fusion events, allowing cytokine uptake and subsequent interaction with nuclear receptor pathway components [13].

There are stem-cell studies where cytokines have been quantified and compared over time, during different states of differentiation, including after treatment with chemicals or growth factors [6,14,15,16]. However, there have been limited studies on the site-to-site variation of cytokine expression from ADSCs isolated from distinct and separate areas from the same donor.

This study had the serendipitous access to donor waste lipoaspirate material from three distinct sites (abdomen, thigh, and chin) from a routine liposuction. The aim of this case study was to investigate any similarities and differences between ADSCs from each location where their cytokines were analysed in three subsample category types: (1) intracellular, (2) secreted, and (3) extracellular vesicle content. Understanding the niche local environment differences of ADSCs, their secretions, and EVs may allow for a greater understanding of the effects that cytokines have on local tissues, as well as their role in signalling and the in situ regenerative capacity of stem cells.

## 2. Results

### 2.1. Live Cell Microscopy

**Figure 1:**
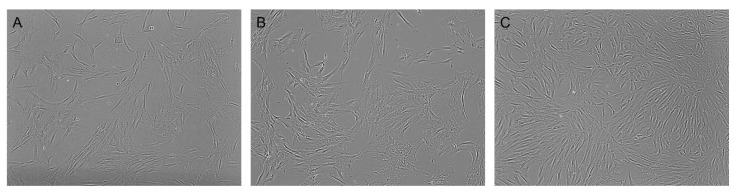
Live cell microscopy showing general morphology of the stem cells isolated from three distinct locations: (**A**) abdomen, (**B**) thigh, and (**C**) chin. All cells were isolated using the same process and plated into culture flasks at an equal density of 1000 cells/mm^2^. Cells were cultured for 336 h with growth medium changed at 84 h intervals. Live images were captured on an EVOS2 prior to harvest. The abdominal (**A**) and thigh (**B**) cultures yielded equivalent cell numbers and percentage confluency at the final timepoint, whereas the chin (**C**) cultures showed an average 10% higher final timepoint percentage confluence.

**Figure 2:**
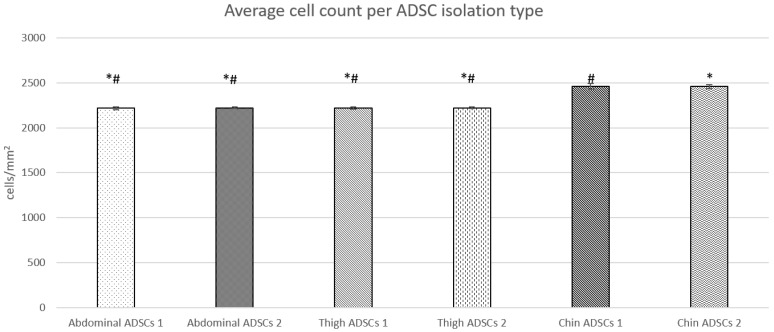
Average cell count across each ADSC isolation type in biological replicates. Abdominal ADSCs and thigh ADSCs shared a similar average count, whereas chin ADSCs showed an average of 10% more cells with no significant difference between each isolation. Student’s *t*-test was performed between all sample types in a single-tail homoscedastic test, where the *p*-value is presented as * *p* < 0.05 and # *p* < 0.05 compared to Chin ADSCs 1 (*) and Chin ADSCs 2 (#). This shows that both abdominal and thigh ADSC isolations had a significant statistical difference in average cell count when compared to both chin ADSC isolations average cell counts.

Live cell microscopy can reveal several qualitative points of information about a cell population’s general health and growth pattern by examining visual checks in morphology, cell number, and cell density. All three subsamples derived from abdominal, thigh, and chin lipoaspirates were processed using the same protocol and plated at 1000 cells/mm^2^ in separate culture vessels. Typically, adipose-derived stem cells present a large, flat, usually bipolar, spindle-shaped or fibroblastic-like morphology (Figure 1A–C). All cells had characteristic ADSC growth patterns and doubling times. This yielded cell populations that were nearly indistinguishable from each other at a glance. Cell shape, size, and general morphology across all three populations were equal. The only standout feature detected was the ADSC-chin population yielding more cells at the final timepoint, showing on average 10% higher confluency than ADSC-abdominal and ADSC-thigh populations (Figure 1C). A Student’s *t*-test (Figure 2) on average cell count between biological replicates of each isolation showed no statistical differences between abdominal ADSC and thigh ADSC numbers; however, a statistical significance in average cell count was identified when compared to the chin ADSC isolations. The chin ADSCs, whilst plated at the same density and cultured in parallel for the same length of time as the other isolations, produced more cells at the experimental endpoint prior to harvest.

### 2.2. Heatmap and Euclidean Clustering of Measured Cytokines

**Figure 3:**
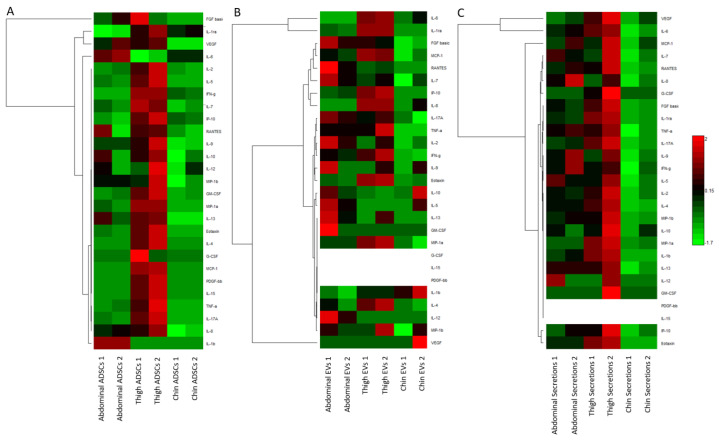
Bioplex quantified cytokines from (**A**) ADSCs derived from abdomen, thigh, and chin; (**B**) EVs produced from ADSCs derived from abdomen, thigh, and chin; (**C**) secretions produced from ADSCs derived from abdomen, thigh, and chin isolations. Log_10_ scale where red is relatively high, green is relatively low, and white represents no values as cytokines were below the level of detection.

From each ADSC isolation (abdominal, thigh, and chin), three subsample categories were derived, i.e., cellular samples, extracellular vesicles (EVs), and secretions. Cytokine expression from each subsample was analysed using the bioplex 27-plex human proinflammatory kit to quantitatively measure 27 distinct cytokines simultaneously in each sample for comparison. To clarify and summarise the complex datasets, three Euclidean clustering dendogram and heatmap images were generated (Figure 3) to examine the cytokine changes in cellular samples (Figure 3A), EVs (Figure 3A,B), and secretions (Figure 3A,C). In a general summary, the heatmaps show there are distinct variations in cytokine content among the three ADSC isolation samples, as well as further variation in content and concentration in the subsample categories of cellular samples, EVs, and secretions.

#### 2.2.1. Cellular Cytokines

Cytokines measured in cells (Figure 3A) from ADSCs derived from abdominal, thigh, and chin lipoaspirates presented consistency between replicates across the majority of measured cytokines. There was, however, a distinct pattern present for each ADSC isolate type. The abdominal ADSC sample cytokine panel shared a relatively median distribution across all cytokines except in IL-1b and IL-6. Comparatively, the closest clustering similarity occurred between abdominal and chin ADSC isolates, whereas the thigh ADSCs show much higher concentrations across all cytokine types except IL-1b and IL-6, which were markedly lower than abdominal and chin ADSCs. Conversely, IL-10, FGF-b, and VEGF shared a closer expression pattern in abdominal and thigh ADSCs. The dendrogram showed six distinct clusters of similar expression patterns across all cell sample types.

#### 2.2.2. EV Cytokines

EVs (Figure 3B) also had distinct patterns emerge that were not too dissimilar from the parent cells. The very apparent observation was no recorded values for G-CSF, IL-15, and PDGF-bb which were below the detection limit within all EV cohort samples. Thigh EVs showed an overall higher cytokine content similar to the cellular heatmap. The abdominal EVs, however, strayed from median to higher content comparatively to their cellular counterparts relative to other EVs. The chin EVs also showed a similar trend to their cellular parent, with a notable variation in VEGF and IL-10. The overall dendrogram clustering showed 7 groupings, not including the three cytokines below detection.

#### 2.2.3. Secretion Cytokines

Secretions had a closer resemblance to the cellular cytokine trends (Figure 3C). The standout cytokines were those that returned not recorded values, where IL-15 and PDGF-bb were again below the detection limit within the EV sample cohort similarly to the EVs. Interestingly G-CSF did have sufficiently detected concentrations with the thigh cohort secretions, appearing amongst the higher recorded cytokine concentrations for the secretion sub-cohort. General trends for the secretion sub-cohort followed similar pattern to the ADSCs; however, the dendrogram clustering pattern appeared to be unique with six groups, not including the values below detection for IL-15 and PDGF-bb.

### 2.3. Parallel Coordinates

**Figure 4:**
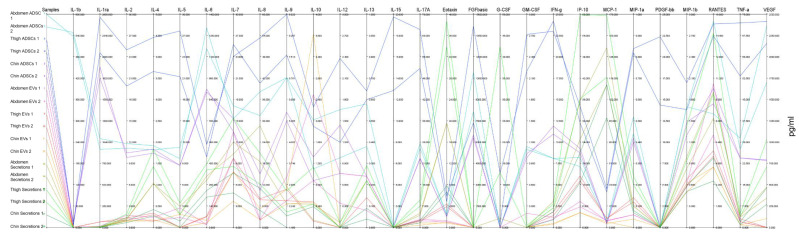
Parallel coordinates plot of Bioplex-27plex cytokine measure with all cytokines on unique linear *y*-axis and each sample denoted by coloured line travelling through its measured cytokine concentration in pg/mL. Abdomen ADSCs (turquoise), thigh ADSCs (blue), chin ADSCs (purple), abdomen EVs (pink), thigh EVs (orange), chin EVs (yellow), abdomen secretions (gold), thigh secretions (light green), chin secretions (dark green).

Cytokine analyses for this study yielded a high-dimensional dataset in which the use of parallel coordinates to visualise a non-scaled output provided a deep analysis with an alternative graphical representation. Figure 4 represents all samples collected (from abdomen, thigh, and chin), as well as subsamples of cells, EVs, and secretions, against all 27 cytokines in a single composite graph. Each cytokine is presented on its own *y*-axis in pg/mL each with its own upper and lower limits while each sample type line is coloured. To reduce visual complexity, biological replicates were group-coloured. This representation allows for all samples to be directly compared without skewing of any axes for cytokines in compilation, where trends of low-expression cytokines could be suppressed by those with substantial concentrations. This analysis validates trends observed in the subsample heatmap and dendrogram of cells, EVs, and secretions. IL-1b and IL-6 were substantially higher in measured concentration in abdominal ADSCs compared to all other sample types. Conversely IL-1ra was almost doubled in thigh ADSCs compared to the abdominal counterpart, while also dominating in terms of IL-2, IL-4, IL-5, IL-7, IL-8, IL-9, IL-12, IL-13, IL-15, and IL-17A. The trends in cellular samples flexed between dominating cells in quantitative measures for most cytokines; however, the comparative trends in EVs and secretions allowed cross-sample trend changes to be viewed. Important noted changes in sample trends are referred to here as ‘breakthrough’ samples, where the measured cytokine appears higher than usual cellular trendsetters. EV breakthrough samples were chin EVs and thigh EVs for IL-10. Secretion breakthroughs were thigh secretions in Eotaxin, G-CSF, IP10, MCP-1, and RANTES. Other important samples were those that appeared as “mid-tier” changes, breaking away from similar sample types but remaining lower than the dominant measures. Notable mid-tier changes were in abdomen EVs IL-7, thigh secretions IL-4, and abdomen secretions for Eotaxin and MCP-1. “Drop-offs” were those sample cytokines that reduced to the lowest point. IL-12, IL-15, Eotaxin, FGF-basic, GM-CSF, MCP-1, PDGF-bb, and TNF-α exhibited several drop-offs across samples. An inverse correlation appeared between some breakthrough and drop-off samples.

### 2.4. Critical Cytokine Content Comparison

**Figure 5:**
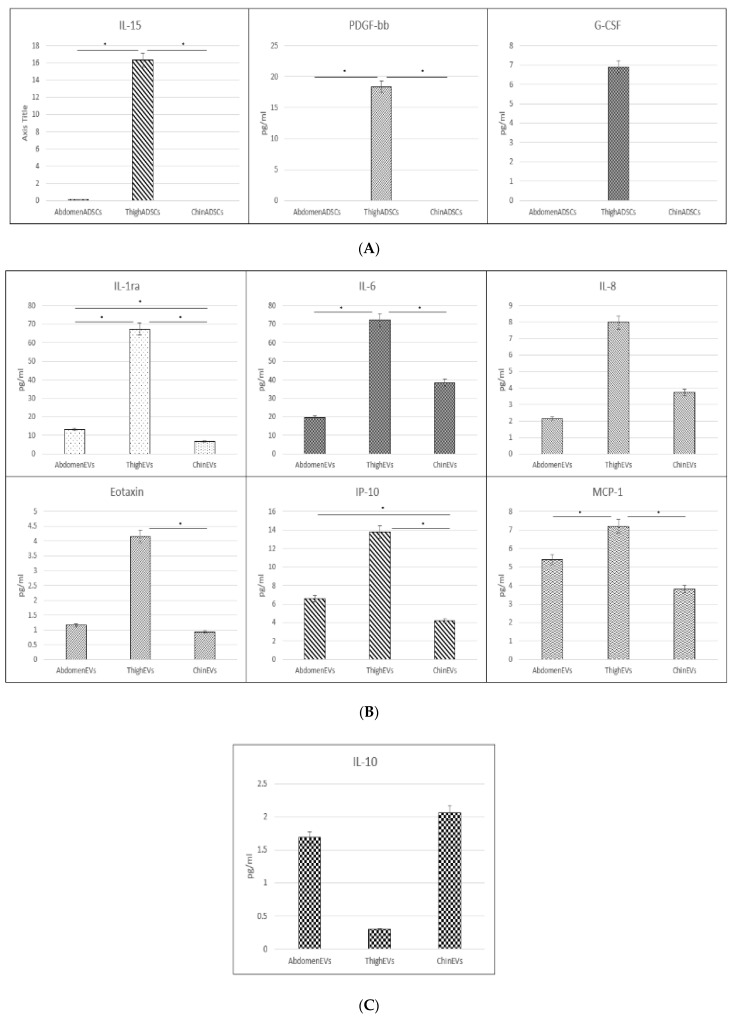
Critical cytokine content comparison graphs: (**A**) ADSC cytokine content of IL-15, PDGF-bb, and G-CSF from ADSCs with the highest measures in thigh ADSCs; (**B**) EV cytokine content for IL-1ra, IL-6, IL8, Eotaxin, IP-10, and MCP-1 with the highest measures in EVs; (**C**) IL-10 content with the highest measure in chin EVs. * *p* < 0.05 according to Student’s *t*-test.

A broad trend across the measured cytokines from intracellular samples saw a general dominance in the expression of cytokines collected from thigh ADSCs. There were, however, three cytokines that stood out, even amongst all the other high measures, i.e., IL-15, PDGF-bb, and G-CSF with 16-, 18-, and sixfold higher averages, respectively, than their abdomen and chin ADSC counterparts which had negligible expression across biological replicates (Figure 5A). These three cytokines had values lower than the detection limit for the EVs and secretions except for G-CSF in thigh secretions, which were 3.8-fold higher than the cellular counterpart. Statistical significance was determined by Student’s *t*-test with *p*-values less than 0.05 in IL-15 and PDGF-bb between thigh ADSCs and abdomen and chin ADSCs, respectively. Conversely, IL-1b and IL-6 were highly expressed in the abdominal ADSCs with IL-1b expression 500-fold higher on average than the other cellular counterparts. Abdomen ADSC IL-6 presented with more than double the average concentration in thigh ADSCs.

In terms of the EV cytokine content (Figure 5B), thigh EVs had six prominent cytokines (IL-1ra, IL-6, G-CSF, Eotaxin, IP-10, and MCP-1) which all followed a similar pattern cluster. Interestingly, the IL-6 trend did not follow the cellular or secretion pattern; in EVs, the content was comparatively higher than its counterparts and cross-measures. On the other hand, Eotaxin trends and the average concentration content between cells and EVs were not too dissimilar, whereas Eotaxin was an order of magnitude higher in secretions across all three sample types. In a reversal of trends, abdomen EV content showed a variant cluster with higher comparative cytokine content in IL-7, RANTES, FGF-basic, IL-9, IL-2, and IL-17A. While the measured cytokine content in EVs did not exceed the concentrations measured in cells, except for the breakthrough chin EV IL-10, the others shared patterns with parent samples. IL-7 and RANTES concentrations in cells, EVs, and secretions were similar. IL-2 and IL-9 EV concentrations were only analogous to secretion samples. While FGF-basic was higher in EV samples, it was dwarfed by the intracellular content, which was more than 1000-fold higher in cells. Statistical significance according to a *p*-value less than 0.05 appeared in all the above cytokines except in IL-8 with significance varying across thigh, abdomen, and chin EVs.

### 2.5. Cytokine Interaction Network Mapping

**Figure 6:**
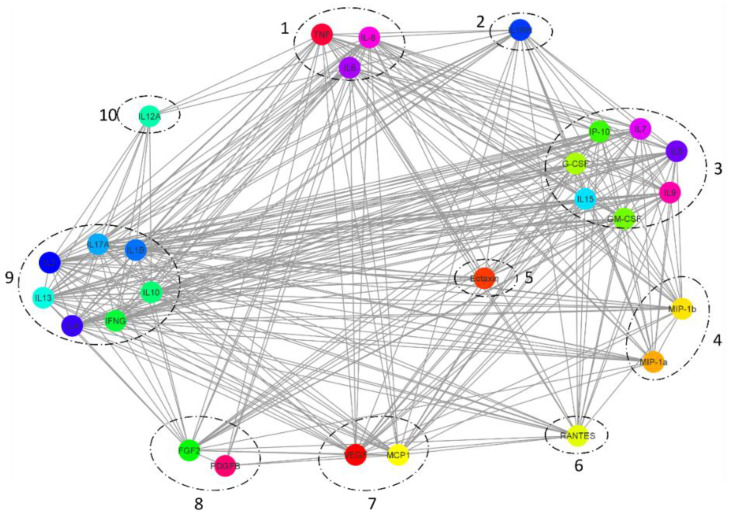
Cytokine interaction network for 27-plex, mapping high interactivity among IL-1b, IL-1rn, IL-2, IL-4, IL-5, IL-6, IL-7, IL-8, IL-9, IL-10, IL-12, IL-13, IL-15, IL-17A, Eotaxin, FGF-basic, G-CSF, GM-CSF, IFN-γ, IP-10, MCP1, MIP-1a, MIP-1b, PDGFB, RANTES, TNF-α, and VEGF, with 307 interactions and an average of 22.7 interactions per cytokine. Ten distinct groups are circled on the basis of the interactivity similarity of node clusters across the network.

A cytoscape protein interaction network (Figure 6) was generated to analyse the interactivity among cytokines. Figure 5 shows each of the 27 measured cytokines (IL-1b, IL-1rn, IL-2, IL-4, IL-5, IL-6, IL-7, IL-8, IL-9, IL-10, IL-12, IL-13, IL-15, IL-17A, Eotaxin, FGF-basic, G-CSF, GM-CSF, IFN-γ, IP-10, MCP1, MIP-1a, MIP-1b, PDGFB, RANTES, TNF-α, and VEGF) mapped to a unique colour for each node. The associated annotated biological function for each cytokine is summarised in Table 1. The connecting lines show individual interactions. To summarise the network statistics in an undirected organic algorithm layout, the number of nodes (cytokines) was 27, and the total number of interactions was 307 with an average number of first neighbour interactions of 22.7 per node. The appearance of 10 distinct group clusters based on similar interactivity is summarised in Table 2.

## 3. Discussion

Stem cells are special cells that have an innate ability for continuous proliferation and self-replenishment, and that can develop into a variety of mature cell types across the body [5]. Their communication and differentiation ability to other cells is thought to be partly controlled by their expression and interaction of a complex composition of surface receptors and secreted molecules [17,18]. The surface proteins include a range of cytokine receptors, which are intrinsically part of cell signalling and communication [19,20]. Cytokines drive signal transduction through interaction with surface receptors in a similar manner to immune cells [21]. Stem-cell activation and differentiation processes are also modulated by conventional or canonical cytokine signalling pathways [22]. Stem cells not only receive cytokine signalling but can also express cytokines as an essential function between cell populations or in response to an external stimuli [6].

The mechanism for cytokine expression has primarily been viewed as a form of direct secretion of molecules through active transportation [8]. However, there are multiple other forms of active transport that include protein pumps, liposomes, and importantly extracellular vesicles [23]. Extracellular vesicles (EVs) have gained substantial traction in recent years due to the cargo they envelope and transport between cells [24]. Recent studies have indicated the EV cargo is selectively packaged and not a random grouping of local molecules as a waste removal mechanism [10,11].

This study investigated intracellular content, direct cellular secretions, and EV cargo from ADSCs derived from three distinct locations: the abdomen, thigh, and chin. The resultant cells were cultured and investigated by live cell microscopy, cytokine assays, and bioinformatics analysis. The cytokines quantified from each cell type and subsample expand our understanding of the differences in the roles these cells may play in local environments and in signalling.

### 3.1. TNF-Alpha, IL-6, and IL-8 Biological Effects in MSCs

Cellular communication is a crucial biological feature ensuring homeostasis, and appropriate cellular responses are triggered in changed conditions such as disease. Previously established cytokines are key components for cellular communication while also having a wide array of biological roles and functions across organs, tissues, and cell types. Adipose tissue is a complex endocrine organ comprising a heterogenous mixture of cells capable of producing a wide range of biologically important molecules. Proinflammatory cytokines have a strong control on organ inflammation at high concentrations [1]. The inflammatory cytokine TNF-alpha is responsible for acute inflammation and signalling that initiates necrosis or apoptosis with importance during resistance of infections and cancer development [25]. It is also well documented that increased adipocyte leads to over production of TNF-alpha, instigating insulin resistance and diabetes [26]. Interestingly, TNF-alpha expression in this study’s thigh ADSCs was more than double the abdomen ADSC and chin ADSC levels. Concomitantly, the measured concentration of IL-8 followed a similar trend to TNF-alpha. In contrast, IL-6 had an inverse trend to TNF-alpha and IL-8 when measured in the intracellular ADSC content. These three cytokines make up the first cluster, interacting with each other, as well as having the unique classification of interacting with all other cytokines within this study, as indicated by the interaction network.

As a pleiotropic molecule, a duality exists where TNF-alpha can promote proliferation or inhibit cellular growth in cancer cells [27]. This is also evident in several stem-cell studies [28,29]. There are two key factors that affect these complex relationships; the first is the available concentration of TNF-alpha expressed, and the second is the protein interactions they encounter. This may be an important element within the local area of the thigh due where a higher inflammatory condition may occur with the natural physical and mechanical stress in the working muscle tissue. A controlled turnover and repair of cells would require an adequate signalling mechanism. In MSCs, it was found that the interaction between TNF-alpha and RUNX2 would directly influence the rate of MSC proliferation, whereby, when RUNX2 was lower, a higher proliferation rate would be observed. This is supported by the observation in adult neural stem cells where RUNX2 is substantially lower than osteogenic differentiating MSCs [30]. RUNX2 and TNF-alpha expression is also observed to be increased in early skeletal muscle and committed bone development [31]. The mechanism of TNF-alpha interaction with cell surface receptors mediates the mitogen-activated protein kinase (MAPK) signalling pathway. MAPK has a sizeable influence and interaction with the extracellular signal-regulated kinase (ERK) and the cJun NH2-terminal kinase (JNK) cascades [32]. The modulation of these pathways controls the cell cycle and proliferation rate. In the MAPK signalling cascade, a differential activation of during MKK3 can drive p38 MAPK activation, which in turn regulates RUNX2 expression that limits proliferation with interaction with TNF-alpha [33]. This plays a dual role both up- and downstream of signalling cascades and possesses feedback control for cellular expression.

IL-6 is a cytokine with both pro- and anti-inflammatory properties. Its multifunctionality imbues roles in normal cellular function, differentiation, migration, and proliferation, along with the ability to instigate certain disease states. Increased IL-6 production and prolonged elevated levels have also been linked to insulin resistance [34]. This notion is supported by the endocrine studies suggesting that excess abdominal adipose tissue increases the chance of insulin resistance. The roles it plays are linked to the interactions it has with cells through signalling processes. IL-6 signalling via the JAK/STAT pathway has a variable control on MSC differentiation or proliferation. Studies have confirmed that IL-6 expression is higher in undifferentiated MSCs, playing a role in their maintenance and proliferation [16,35]. In classical signalling, IL-6 has regenerative and anti-inflammatory functions, which are enacted through STAT3 activation of the signalling pathway via the surface IL-6 receptor [36]. However, for an anti-inflammatory function, a trans-signalling processes is achieved via a soluble receptor gp130 [37] or through active IL-6 uptake linked to EV packaging and translocation in cells that lack an IL-6 receptor [38]. In this study, the IL-6 thigh ADSC EV content ratio was substantially higher than that in abdomen ADSCs and chin ADSCs. Furthermore, the IL-6 thigh ADSC EV and secretion content showed an inverse trend to cellular content. The stark difference in sample type EV content compared to cellular content alludes to a controlled cargo packaging difference between the cell types. The proliferative capacity of MSCs is also maintained by IL-6 regulation of the ERK1/2 pathway [39], downstream of MAPK, dually interacted on by TNF-alpha. Complementary interaction of IL-8 in the activation of ERK1/2 through MAPK signalling has also been implicated in promoting the proliferative capacity of undifferentiated MSCs by increasing the expression of VEGF [40,41].

While it is clear TNF-alpha, IL-6, and IL-8 are highly multifunctional across different cells, the complexity of cytokine roles is slowly still being unravelled, particularly the effect of EV content and its role in signalling and cellular proliferation.

### 3.2. Critical Cytokine Content Changes in Cells

IL-1b in abdominal ADSCs presented with the single highest ratio difference among all other sample types. There were indeed higher ratio differences, i.e., IL-6 and FGF; however, they occurred for all sample cell types. IL-1b was unique being orders of magnitude higher than the next cell or subsample type with both a biological and a statistical significance. The biological relevance of IL-1b is a potent proinflammatory cytokine which induces B-cell activation and antibody production. It also has a strong influence on cell proliferation. Some studies on the effects of IL-1b on MSCs have shown that IL-1b pre-treatment of ADSCs enhanced the repair of injured intestinal cells [42]. In a study by Sullivan et al., it was found that IL-1b and TNF-alpha influenced the differentiation and migration of murine MSCs independently of the NF-κB pathway [43]. None of the other interaction network clustered group 9 cytokines or dendrogram alignments followed the expression levels or trends of IL-1b. An antagonistic relationship between IL-1b and IL-1ra has been linked to immunosuppressive effects in MSCs [44]. However, no correlations could be drawn between these two cytokines in this study as there was an equally high level of IL-1ra across all intracellular ADSCs.

PDGF-bb, IL-15, and G-CSF in the thigh ADSCs were observed as breakthrough cytokine changes, displaying higher levels in thigh than any other ADSC sample. PDGF-bb from group 8 had the relatively fewest overall interactions within this study, interacting with only six other cytokines (TNF-alpha, IL-6, IL-8, FGF2, VEGF, and MCP-1), most of which have important roles in signalling and proliferation. PDGF-bb is a growth factor that plays an essential role in the regulation in development, cell proliferation, cell migration, survival, and chemotaxis [45]. It is a potent mitogen for mesenchymal cells [46]. PDGF-bb-mediated signal transduction is activated via the PI3K pathway by phosphorylation of the PDGF receptor; alternatively, signal transduction is also activated by selective inhibition of STAT3 [47]. These downstream modulations can regulate the cell cycle and proliferation of mesodermal cells [48]. The increased expression in the thigh ADSCs could indicate a higher turnover and migratory pattern of cells in a highly used and muscular region over the abdominal and chin area.

The remaining breakthrough cytokines IL-15 and G-CSF were from group 3 and had no direct interactions with PDGF-bb. Their similar expression patterns across cells, EVs, and secretions may have a valuable biological implication. In the thigh ADSC samples, their levels were voluminously higher than its counterparts for both cytokines. Canonically, IL-15 is known to stimulate the proliferation of T lymphocytes in adaptive immunity. Fascinatingly, it was also found to regulate proliferation and self-renewal of adult neural stem cells as a key mechanism for JAK/STAT and ERK pathway activation [49]. The expression of IL-15 in substantial concentrations at one site, thigh ADSCs, compared to others may indeed be a microenvironment niche response. The base functionality of these cells across the body plays a similar role, but may be more niche-specific in localised areas. This notion is supported by the study by Cui et al. [50], in which IL-15 was found to be differentially expressed in distinct subsets of stromal cells akin to this study.

Similarly, G-CSF, expression follows trend of the aforementioned cytokines in ADSCs. It has a well-known role in the immunomodulation of granulocytes. However, the niche notion may coincide with location role variation as a recent study found that G-CSF in ADSCs can modulate the cell mobility and metabolism of hyaluronan [51]. This is supported by the local functionality variation where working muscle regions have a higher local resting and active metabolic rate over the abdominal region, which is rather considered an endocrine region. Furthermore, G-CSF has a complex relationship with Jak/STAT, Ras/MAPK, and PI3K, and its expression is controlled by IL-1b and TNF-alpha in two separate sources of MSCs [7].

### 3.3. Extracellular Vesicle Cytokine Content

While cytokines IL-15, PDGF-bb, and G-CSF have closely related trends in cells, secretions, and EVs, there are individual cytokines in other groups that share a similar trend in EV content. The varied collection of cytokines includes group 2 IL-1rn, group 5 Eotaxin, group 3 IP-10, and group 7 MCP-1. These molecules have diverse base functions and roles in response to disease. However, a shared feature amongst them is in the recruitment of specialised immune cells. Their functionality as chemotactic molecules provides commonality in cell–cell communication. In inflammatory circumstances, these molecules are secreted from cells to mediate the migration of immune cells toward the site of interest. This process is facilitated through receptor interaction. The presence of chemotactic molecules enveloped within extracellular vesicles may have less of a chemoattractant property as a bypass of surface receptors on immune cells, thus increasing the chance of EV interaction with other cells through membrane fusion events. This would also allow for a possible increase in bioavailability to be transferred further than local environments. The impact for cross-cell communication without stimulating chemotaxis is an interesting mode, which could be used more in alignment with intracellular signalling or to produce similar or other cytokine molecules. IL-1rn functions primarily as an IL-1 receptor antagonist, which is a surface receptor; however, intracellularly, IL-1rn can interact with several protein partners, but the biological roles are still undefined. Eotaxin on the other hand is an eosinophil attractant; however, it can induce phosphorylation of MAP3K, leading to kinase activation in the MAPK signalling pathway [52]. Similarly, MCP-1 also induces activation of MAPK via ERK [53]; however, it has a unique ability to induce the production of adhesion molecules [54]. These mechanisms have been minimally investigated but hold an interesting biological function to be explored.

The final cytokine IL-10 was the only breakthrough cytokine to have a higher measured concentration than all other samples in chin-EVs. It has a fascinating role as a cytokine synthesis inhibitory factor, inhibiting the production of IFN-gamma, IL-2, IL-3, TNF, and GM-CSF [55], thus reducing an immune response or counteracting the production of proinflammatory molecules. Its presence in this location at a very high level only within EVs indicates that it may not be performing its primary role as an anti-inflammatory cytokine. It has been noted to intracellularly activate Jak1 and Tyk2 in the JAK/STAT signalling cascade; the activation of Tyk2 controls the activation of STAT3 [56], which is known to mediate gp130 cell cycle and proliferation [57].

In conclusion, this study is unique in its examination of three different site-specific stem-cell isolations, with a focus on cytokines from cellular samples, secretions, and extracellular vesicles. Analyses explicated previously unknown differences between ADSCs cytokine expression and contents between these locations and in all fractions. There is a clear correlation that cytokine content, concentration, and importantly location are an integral part of biological molecular mechanisms. The complex interactions, balances, and counterbalances of cytokines in their pathway signalling cascade involvement have several layers of redundancies to maintain an equilibrium and regulate cellular states while oscillating multifunctionality. Furthermore, communication layers beyond canonical signalling are at play for complex interactions and pathway modulation between cells using an arsenal of subcellular mechanisms. While there are very limited studies examining cytokines in ADSCs difference in niche locations and their derived EVs, this study provides a new avenue for research to be conducted on the biological effects of EV encapsulated cytokines on stem-cell signalling. Further time-dependent studies that investigate changes in concentration correlative to location and health status may provide a wealth of information. This will impact the field, ensuring that studies are comparing like cells to one another. This may also motivate new research into therapeutic opportunities.

## 4. Materials and Methods

### 4.1. Sample Collection

The procedures of adult human ADSCs isolation were adopted from Santos et al. [14] with approval from UTS-HREC Santos-2013000437. Donor participants volunteered material through informed consent for waste lipoaspirate donation as per ethics guidelines and were deidentified for research purposes. A total of six separate sample tubes were provided: two from abdomen, two from thigh, and two from chin. Collection was completed at Matraville Medical Complex by medical staff; all samples were collected on the same day and processed simultaneously.

### 4.2. Cell Culture

Cell expansion procedures were adopted from Santos et al. [14]; ADSCs (abdomen, thigh and chin) in replicates were seeded at 1000 cells/mm^2^ in technical triplicates in separate T175 flasks (Nunc, ThermoScientific, Carlsbad, CA, USA) in a medium mixture comprising DMEM Glutmax/F12 (Gibco, Life Technologies, Carlsbad, CA, USA) with 10% foetal bovine serum (FBS, Gibco, Life Technologies, Carlsbad, CA, USA) incubated at 37 °C at 5% CO_2_. ADSC medium was aspirated and replaced every 84 h for a total of 14 days. Then, cells were harvested at sub-confluence post EV and secretion collection, by washing adhered cells in PBS and then stripping with TrypLE Express (12604 Gibco). Cell counts were completed using the Countess 2 (Thermo Fisher Scientific, Sydney, NSW, Australia) according to the manufacturer’s guidelines. Harvested cells were then stored at −80 °C until sample preparation.

### 4.3. Extracellular Vesicle Isolation

EVs were isolated similarly to the procedure outlined in Dalla et al. [11], where growth medium was collected from each ADSC cell sample (abdomen, thigh, and chin), and EVs were isolated by differential centrifugation. Media from each cell sample was centrifuged at 20,000× *g* for 1 h at 4 °C to pellet EVs. The pellet was then resuspended in 1× sterile phosphate-buffered saline (PBS) (Sigma-Aldrich, Sydney, NSW, Australia) and centrifuged at 2000× *g* for 1 min to remove debris. The supernatant was centrifuged again 22,000× *g* for 30 min at 4 °C to pellet EVs. The EVs were resuspended in PBS and stored at −80 °C until sample preparation. Concentrations of EVs were determined and normalised by protein content using the Qubit protein assay (Thermo Fisher Scientific, Sydney, NSW, Australia) following the manufacturer’s protocol.

### 4.4. Secretion Isolation

The growth medium from each cell sample was collected post EV isolation; once EVs were pelleted, 500 μL of supernatant was collected from each sample and stored at −80 °C until sample preparation.

### 4.5. Sample Preparation

All samples were retrieved from −80 °C storage and thawed on ice. Samples were in biological replicates, denoted as abdomen ADSCs 1 or 2, abdomen EVs 1 or 2, abdomen secretions 1 or 2, thigh ADSCs 1 or 2, thigh EVs 1 or 2, thigh secretions 1 or 2, chin ADSCs 1 or 2, chin EVs 1 or 2, and chin secretions 1 or 2. ADSC and EV samples were prepared in the same manner, where pellets stored in PBS were centrifuged for 10 s at 10,000× *g* and then lysed to release inner cytokines using a probe sonicator (Sonics & Materials, Inc., Newtown, CT, USA) three times with 10 s bursts each while on ice. Lysed ADSCs and EVs were then centrifuged at 20,000× *g* for 10 min to removed debris, collecting supernatant in fresh Eppendorf tubes for analysis. Secretion samples were also centrifuged at 20,000× *g* for 10 min, collecting supernatant in fresh Eppendorf tubes for analysis.

### 4.6. Cytokine Assay

Bioplex analysis was performed as per Santos et al. [6] according to the manufacturer’s guidelines. Generally, 50 μL of the prepared samples from ADSCs, EVS, and secretions final volume were used from each biological replicate to simultaneously determine concentrations of IL-1rn, IL-1b, IL-2, IL-4, IL-5, IL-6, IL-7, IL-8, IL-9, IL-10, IL-12 (p70), IL-13, IL-15, IL-17, Eotaxin, FGF-basic, G-CSF, GM-CSF, IFN-γ, MCP-1, MIP-1a, MIP-1b, PDGF-bb, RANTES, TNF-α, and VEGF, using commercially available multiplex bead-based sandwich immunoassay kits (Bioplex human 27-plex, M50-0KCAF0Y Bio-Rad Laboratories Hercules, CA, USA).

### 4.7. Data Analysis

Data analysis was completed using Microsoft Excel 365, DanteR software (DanteR version 1.0.0.10; R version 2.12.0 The R Foundation for Statistical Computing, Auckland, New Zealand) [21] and Cytoscape (version 3.8.2 Cytoscape Consortium, Seattle, WA, USA) [58].

## Figures and Tables

**Table 1:** Biological annotation for cytokines and chemokines.

Name	Full Name	Accession	Gene	Biological Annotation
IL1b	Interleukin 1, beta	P01584	IL1B	Potent proinflammatory cytokine
IL1rn	Interleukin-1 receptor antagonist protein	P18510	IL1RN	Inhibits the activity of interleukin-1
IL2	Interleukin-2	P60568	IL2	T-cell growth factor
IL4	Interleukin-4	P05112	IL4	Lymphocyte stimulatory factor 1
IL5	Interleukin-5	P05113	IL5	Eosinophil differentiation factor
IL6	Interleukin-6	P05231	IL6	B-cell stimulatory factor 2
IL7	Interleukin-7	P13232	IL7	Hematopoietic growth factor capable of stimulating the proliferation of lymphoid progenitors
IL-8	Interleukin-8	Q9UCS0	CXCL8	Monocyte-derived neutrophil chemotactic factor; IL-8 is a chemotactic factor that attracts neutrophils, basophils, and T-cells, but not monocytes
IL9	Interleukin-9	P15248	IL9	T-cell growth factor P40
IL10	Interleukin-10	P22301	IL10	Cytokine synthesis inhibitory factor
IL12	Interleukin-12	P29459	IL12A	Cytotoxic lymphocyte maturation factor 35 kDa subunit
IL13	Interleukin-13	P35225	IL13	Inhibits inflammatory cytokine production
IL15	Interleukin-15	P40933	IL15	Cytokine that stimulates the proliferation of T-lymphocytes
IL17A	Interleukin-17A	Q16552	IL17A	Cytotoxic T-lymphocyte-associated antigen 8
Eotaxin	Chemokine (C-C motif) ligand 11	P51671	CCL11	In response to the presence of allergens
FGF-basic	Fibroblast growth factor 2 (basic)	Q9UCS5	FGF2	Plays an important role in the regulation of cell survival, cell division, angiogenesis, cell differentiation, and cell migration.
G-CSF	Granulocyte colony stimulating factor 3	P09919	CSF3	Granulocyte/macrophage colony-stimulating factor
GM-CSF	Granulocyte-macrophage colony stimulating factor 2	P04141	CSF2	Cytokine that stimulates growth and differentiation
IFN-γ	Immune interferon gamma	P01579	IFNG	Produced by lymphocytes activated by specific antigens or mitogens
IP-10	10 kDa interferon gamma-induced protein	P02778	CXCL10	Chemotactic for monocytes and T-lymphocytes
MCP1	Monocyte chemotactic and activating factor	P13500	CCL2	Chemotactic factor that attracts monocytes and basophils but not neutrophils or eosinophils
MIP-1a	Macrophage inflammatory protein 1-alpha	P10147	CCL3	Monokine with inflammatory and chemokinetic properties
MIP-1b	Monocyte adherence-induced protein 5-alpha	Q8NHW4	CCL4L1	Chemokine that induces chemotaxis of cells expressing CCR5 or CCR1
PDGFB	Platelet-derived growth factor beta polypeptide	P01127	PDGFB	Growth factor that plays an essential role in the regulation of embryonic development, cell proliferation, cell migration, survival, and chemotaxis
RANTES	Eosinophil chemotactic cytokine	P13501	CCL5	Chemoattractant for blood monocytes, memory T helper cells, and eosinophils; causes the release of histamine from basophils and activates eosinophils
TNF-α	Tumour necrosis factor ligand superfamily member 2	P01375	TNF	Cytokine that binds to TNFRSF1A/TNFR1 and TNFRSF1B/TNFBR
VEGF	Vascular endothelial growth factor A	Q9H1W9	VEGFA	Growth factor active in angiogenesis, vasculogenesis, and endothelial cell growth

**Table 2:** Cytokine grouping based on cluster interactivity similarity across network, showing 10 distinct groups with various levels of directed and noninteraction with other group cytokines.

Group	Cytokines in Group	Number of Cytokines in Group	Interaction Description	Affiliates/Non-Affiliates
1	TNF, IL-8 and IL-6	3	Interacts with all groups	Groups 1–10
2	IL1rn	1	Does not interact with	Group 8 Group 10
3	IL-5, IL-7, IL-9, IL-15, GM-CSF, G-CSF and IP-10	7	Does not interact with	Group 10 PDGFB from group 8
4	MIP-1a and MIP-1b	2	Does not interact with	Group 2 Group 5 Group 8 Group10
5	Eotaxin	1	Does not interact with	Group 4 Group 6 Group 7 Group 10 PDGF from group 8
6	Rantes	1	Does not interact with	Group 10 MIP-1a from group 4PDGF from group 8
7	MCP1 and VEGF	2	Does not interact with	Group 5 Group 10
8	FGF2	2	Does not interact with	Group 2 Group 10
	PDGF		Does not interact with	Group 2 Group 3 Group 4 Group 5 Group 9 Group 10
9	IL-2, IL-17A, IL-1B, IL-10, IFN-γ, IL4, and IL13	7	Does not interact with	PDGF from group 8
10	IL-12A	1	Interacts only with	Group 1 Group 9 Group 10

## Data Availability

All important data is included in the manuscript.
